# Reflections on ‘Have we lost sleep?’

**DOI:** 10.1017/mdh.2024.20

**Published:** 2024-07

**Authors:** A. Roger Ekirch

**Affiliations:** Virginia Tech, History, Blacksburg, VA, USA

**Keywords:** Segmented sleep, Western world, Pre-industrial, History

## Abstract

The article, ‘Have we lost sleep? A reconsideration of segmented sleep in early modern England’, *Medical History*, 67, 2 (2023), 91–108, by Niall Boyce is devoted to criticising my historical research pertaining to 1) the predominance of segmented sleep in the pre-industrial Western world and 2) the nineteenth-century transition of sleep to today’s pattern of continuous slumber that most people in modern societies seek to achieve, albeit not always successfully. This response addresses Boyce’s reinterpretation of the evidence and indicates whether this is erroneous or selective. My analysis thereby reasserts the predominance of segmented sleep in pre-modern Western Europe. Boyce’s assessment rests not on his original investigation of primary sources but on my first study relating to segmented sleep, published in 2001. Not least of the flaws of ‘Have We Lost Sleep?’ is its surprising inattention to my subsequent works that have expanded, modified, and bolstered this initial publication.

My critique of the article ‘Have we lost sleep? A reconsideration of segmented sleep in early modern England’, *Medical History*, 67, 2 (2023), 91–108, by Niall Boyce begins by addressing several broad issues.^1^ This is followed by a discussion of specific instances in which he has misconstrued references drawn from primary sources. At the end of the article, I offer several methodological observations.

Boyce’s assessment of my research rests not on his rigorous investigation of primary sources but on my first publication relating to segmented sleep (also known as biphasic or bimodal sleep) in early modern Britain and continental Europe. Published in 2001, in *The American Historical Review*, ‘Sleep We Have Lost’, I like to think, has aged well. A voluminous quantity of evidence that has since emerged has only strengthened my conviction that the predominant pattern of Western sleep in the pre-industrial world was segmented, whereby most individuals usually retired between 9 and 10 p.m., awakened from their ‘first sleep’ about midnight or later, remained awake on average for up to an hour or so, and then took a ‘second sleep’ roughly until dawn.^2^ Allusions to the ‘first’ and ‘second sleep’ are plentiful in early modern texts and are also well-represented in medieval literature and, though fewer in number, in such classical works as Livy’s *History of Rome* (27–9 BC) and Plutarch’s *Life of Themistocles* (75 AD).^3^ In the first century BC, Virgil in the *Aeneid* wrote of the ‘hour which terminates the first sleep, when the car [chariot] of Night had as yet performed but half its course’.^4^

In the course of publishing the book, *At Day’s Close: Night in Times Past*, in 2005 and three additional articles in recent years in peer-reviewed historical and medical journals, I have modified, expanded, and bolstered a number of points advanced in ‘Sleep We Have Lost’. For example, ‘The Modernization of Western Sleep: Or, Does Insomnia Have a History?’, published in *Past & Present*, examines at length the transition during the nineteenth century from segmented sleep to the consolidated slumber that today those of us in the industrialised world seek to achieve, though not always successfully.^5^ This transition in sleep patterns, which was protracted and uneven, occurred later than I had first speculated in ‘Sleep We Have Lost’. In light of numerous nineteenth-century sources, segmented sleep remained prevalent well into the 1800s.

I am genuinely surprised that Boyce has chosen to all but ignore my later publications in addition to a wealth of new evidence, including more than two hundred excerpts on my university website drawn from novels, letters, poems, essays, travel accounts, newspapers, court records, and periodicals, which are illustrative of segmented sleep that I have not previously quoted in my published work: https://sites.google.com/vt.edu/roger-ekirch/sleep-research/segmented-sleep. Rather than just passing allusions, of which there are upwards of two thousand to segmented sleep found to date (typically phrased in such a way as to suggest that segmented sleep was wholly familiar among contemporaries), most of the ‘website selections’ document the prevalence of this pattern of slumber with additional details. Unfortunately, though the author knew of these selections, they too, like my later publications, received fleeting attention in his article. And yet in evaluating anyone’s scholarly research on a given topic – be that person a historian, a cardiologist, or an engineer – all relevant information, especially the most recent, should be thoroughly examined.

Had Boyce done this, his article would have avoided a number of unfortunate mistakes. He attaches considerable importance, for example, to a book about dreams in which its author ‘identified no single, unambiguous early modern medical discussion of segmented sleep’.^6^ Yet in at least eighteen medical texts published in the seventeenth and eighteenth centuries in England and France, references cite the interval after one’s ‘first sleep’ as a preferred time to ingest pills, potions, and elixirs, some of which first had to be composed with the aid of ‘recipe books’. ‘The jaundice is cured’, instructed the antiquary John Aubrey, ‘by putting the urine after the first sleep, to the ashes of the ash-tree’.^7^ These recommendations represent an early, albeit primitive, form of chronopharmacology. In short, contrary to Boyce’s speculation, ‘first sleep’ did not represent a subliminal phase of continuous sleep.^8^ Customary usage plainly confirms that ‘first sleep’ comprised a distinct period followed by an interval of wakefulness. Typically, descriptions recounted that an aroused individual had ‘taken’, ‘gone through’, ‘awakened from’, ‘had’, ‘gotten’, or ‘come out of’ his or her ‘first sleep’. Early modern European physicians clearly took for granted that people naturally awakened after their ‘first sleep’ before taking a ‘second sleep’ that followed an interval of consciousness of varying length. The advice to ‘lye to sleep again’ was one of various expressions occasionally employed in place of the term ‘second sleep’. Others included ‘morning’, ‘latter’, or ‘last’ sleep. Sometimes, we are merely informed that an individual after awakening from their ‘first sleep’ returned to bed.^9^ Sir Francis Bacon advised that an elixir ‘in the morning be ingested *between sleeps* [my italics]’.^10^

Unfortunately, these instructions do not provide detailed descriptions of segmented sleep, but why would they? Neither is it possible to find detailed discussions in early modern texts of other routine bodily functions, such as urinating, defecating, consuming food, drinking water, and breathing. There *are*, on the other hand, revealing references in medical literature over the span of six centuries that I have found pertaining to sexual intercourse that Boyce ignores. Of a patient in Florence, an alchemist in the twelfth century advised, ‘Let him not abstain entirely from intercourse, but let him enjoy it when it can be very pleasurable and after the first sleep; but let him not engage in it too frequently’.^11^ Noting the large number of children in peasant households, the sixteenth-century French physician Laurent Joubert wrote that bone-weary labourers, too exhausted after a day’s work, engaged in intercourse ‘after the first sleep’ when ‘they have more enjoyment’ and ‘do it better’.^12^ Years later, in 1826, the physician Joseph Briand counselled,

Not every season, every moment of the day is equally conducive to the accomplishment of the conjugal act….The night is generally more suitable than the day, and the moment of waking after the first sleep is preferable at any other hour, because all the organs are then rested of the day’s work and that the rest of the night will be able to repair the forces.^13^

Similar recommendations may be found in my publications and on my website. Given prevailing theories of digestion, texts also urged persons to sleep on one side of the body when first retiring and then, after awakening from their first sleep, to sleep on the other side.^14^

I have come across no references in medical texts to the modern concept of ‘middle-of-the-night insomnia’, also known as ‘sleep maintenance insomnia’, which is a ‘primary insomnia’ for which no explicable reason exists for waking, including physical and mental distress as well as environmental vexations. In contrast, ‘sleep onset insomnia’, occurring at bedtime, generated a variety of remedies in early writings.^15^ Naturally waking up in the middle of the night for no evident reason, however, was thought normal until it became pathologised as a disorder in the late 1800s. By that time, as I have written in ‘The Modernization of Western Sleep’, segmented sleep, owing to cultural and technological consequences of the Industrial Revolution, had given way, for the most part, to the consolidated sleep that most persons in the industrialised world currently aspire to experience. Even so, in a number of cases today, nocturnal wakefulness for some persons, rather than a sleep disorder, likely represents a strong echo or a persistent remnant of this older predominant pattern of slumber,^16^ an interpretation in which a number of sleep scientists have taken an interest. According, for example, to Charles A. Czeisler, the Baldino Professor of Sleep Medicine and the Director of the Division of Sleep Medicine at Harvard Medical School, these ‘findings have led to changes in the practice of sleep disorders medicine, particularly for patients with middle of the night insomnia’.^17^

Another unfortunate consequence of Boyce’s apparent lack of familiarity with my later publications is his wholly inaccurate statement that ‘the scientific and anthropological evidence that Ekirch employs to support the idea of segmented sleep is best described as selective’.^18^ Quite the contrary. With regard to the Tiv people, subsistence farmers in central Nigeria who were the subjects of an anthropological study in the mid-twentieth century, the evidence of biphasic sleep – ‘At night, they wake when they will and talk with anyone else awake in the hut’ – is both compelling and straightforward^19^. More to the point, in ‘Segmented Sleep in Preindustrial Societies’, published in 2016 in the journal *Sleep*, I cited a variety of observations by travellers and anthropologists, from the early modern era to the twentieth century, to demonstrate that an array of non-Western preindustrial cultures, bereft of artificial lighting, experienced a pattern of biphasic sleep,^20^ a conclusion similarly advanced in the research of David Samson and colleagues who studied a rural community in Madagascar – a pattern of sleep, they concluded, ‘strikingly similar to the “first sleep” and “second sleep” pattern described by Ekirch’.^21^ Of the Tupinamba Indians in Brazil, the French priest André Thevet reported in 1555 that they ate whenever they had an appetite, ‘even at night after their first sleep they get up to eat and then return to sleep’.^22^ Or, to take another example, in 1895, a British anthropologist wrote of the Woolwa people in Central America, ‘ Frequently at night, after the first sleep, the men would gather round the fires from their respective quarters in the lodge, and, as they warmed themselves in the flames from the chill of the night air, would enjoy some yarn with a quiet chuckle’.^23^ Noting studies currently underway of South American cultures without artificial light, Russell Foster, the Head of the Sleep and Circadian Science Institute at Oxford, recently observed, ‘We’ve got enough evidence that our natural pattern is not this mythical eight hours of consolidated sleep. It is much more likely to be polyphasic or biphasic’^24^.

With regard to Boyce’s dismissal of Thomas Wehr’s ‘single scientific study’ at the National Institute of Mental Health (96–97),^25^ it and his related articles are regarded by the sleep medicine community as landmark contributions, and they have been extensively cited.^26^ Neither Wehr nor I have claimed that his clinical experiment afforded an ideal setting to probe how humans, in the absence of artificial illumination, slept in the past. Indeed, he has acknowledged obvious differences between his research and pre-industrial social and cultural conditions. But what impressed us both was the impact that light has on the human body clock, particularly on the sleep–wake cycle. Nor is it coincidental that an absence of artificial light, either in past years or in some instances today, has been conducive to segmented sleep. Notably, though Boyce cites the availability of light at night in early modern Western households, primitive sources of illumination such as rushlights, pine knots, oil lamps, and tallow candles, which I describe at length in *At Day’s Close*, were much too weak to have affected the sleep–wake cycle.^27^ Moreover, at the least, it is surprising that having first criticised the value of efforts to recreate conditions of pre-industrial sleep in today’s world, Boyce cites a 2013 study based upon the sleep of eight people during a two-week camping expedition in which a fire supplied their only light at night.^28^ Wehr’s research determined that a span of several weeks without light at night was necessary for a biphasic pattern of sleep to emerge.^29^

The author also speculates that ‘[s]ome material cited by historians in support of routinely segmented early modern sleep might also be read simply as evidence of undesirable broken sleep’.^30^ The tenth and eleventh chapters of *At Day’s Close*, in fact, explore in depth numerous impediments to both the safety and quality of early modern sleep, from illness to environmental annoyances.^31^ But biphasic sleep, in its own right, was not characterised as a source of restless or disturbed sleep, whether in medical texts, in literature, or in the numerous early modern diaries that I have read. Again, this utterly familiar form of slumber was thought perfectly natural. If a diary recorded that a person’s ‘first sleep’ had been disrupted, for example, by a thunderstorm, he or she would write of their ‘first sleep’ having been disturbed by a storm. The diarist Robert Sanderson, who on occasion was awakened prematurely ‘on my first sleep’ by his dog, naturally arose from bed on other nights to sit and smoke a pipe, once after first checking on his ill wife.^32^ Boyce cites the work of Sasha Handley, the author of *Sleep in Early Modern England*, who clearly distinguishes between ‘waking between the first and second sleep’ and ‘accidental waking, which might be due, for example, to the noise of a striking clock’.^33^ With regard to a prayer cited by Boyce there is also a difference drawn in the prayer between ‘in the Night *when you awake, or cannot sleep* [my italics]*’*, a distinction that Boyce recognises but then enigmatically infers that ‘their grouping together within this text suggests that both might be undesirable or even distressing states’.^34^

He alludes in his article to my use of ‘fragments’ and ‘shards’ of evidence of which I have previously written, a practice familiar to historians in addition to relying upon more substantive sources as I and other historians routinely do. In my previous work, I have sometimes drawn an analogy to a jigsaw puzzle. Rather than any single reference providing a highly detailed description of segmented sleep – its stages, dynamics, and timing – collectively, numerous references from primary sources leave no doubt about the major features of this pattern of slumber or its prevalence. As in a jigsaw puzzle of a dog, for instance, the absence of scattered pieces and the worn edges of other pieces cannot be construed to suggest the image of a cat once the puzzle has been completed. Unfortunately, Boyce seems determined, figuratively, to identify either a cat or, more accurately, no image at all, even when an overwhelming number of pieces of this metaphorical puzzle portray a dog.

If only individuals in the past had worn actiwatches permitting us to calibrate their sleep with the precision that Boyce evidently expects^35^ . Ultimately based on some two thousand textual references of varying quality, my best estimate has been that most people, *on average* after their ‘first sleep’, remained awake for up to an hour or so depending on the nature of their activity. Some people no doubt remained awake for shorter or longer stretches, just as some slept for different lengths of time. Contrary to Boyce’s assertion, I have never described any of these intervals as ‘well-defined’.^36^ Little about sleep is quite so tidy. Instead, I emphasised in *At Day’s Close* that people did not sleep according to the same timetable, much less follow a rigid pattern of slumber. In short, not all periods of sleep and wakefulness were the same length, even within the same households, bedchambers, or beds. Bedtimes and rising times could also vary. A French essayist correctly observed in 1752, ‘The time of the first sleep is not the same for everyone’.^37^ In the nineteenth-century French novel, *The Wandering Jew*, a father asks his son, ‘Agricola, are you asleep, my boy? As for me, my first sleep is over. My tongue is devouring me, like the devil, with the desire of talking’.^38^

After all, the existence of miscellaneous variations no more diminishes the pre-industrial predominance of biphasic sleep than variations in modern slumber contradict the widely acknowledged predominance today in Western Europe and the United States, among other locations, of consolidated sleep. *‘*Predominance’, of course, has a distinct meaning quite apart from either ‘universal’ or ‘ubiquitous’.

A small fraction of the population, as I have written, shunned taking a second sleep. My publications and my website provide examples of prominent figures who were admired for choosing to remain awake for the remainder of the night. The Duke of Wellington famously insisted, ‘Have but one sleep, and with the first turn, turn out’.^39^ In contrast, his adversary at Waterloo, Napoleon, on awakening, reportedly ‘explained and directed with a clear head, after his first sleep, all the general arrangements for the coming day; and then, after a second and refreshing repose superintended the execution of them’.^40^ Well known were selected clergymen who chose to reserve the small hours of the morning for prayer and study, only electing to return to bed if overcome by sleep. Such individuals, few in number, represented proverbial ‘exceptions that prove the rule’ by being acknowledged by contemporaries for their industry and dedication in deviating from conventional behaviour.^41^ ‘Early risers’ were sufficiently small in number before the mid-nineteenth century for an English surgeon to marvel in 1828, ‘I have known persons who never indulged in a second sleep’.^42^

Further, I have not suggested that dreams in the ‘first sleep’ were more vivid than in the ‘second’.^43^ What I *have* written, as Wehr has reported from his clinical research, is that his subjects often awakened from ‘vivid’ dreams occurring in the ‘first sleep’, for which I have given a number of examples in my publications drawn from historical sources. I do not claim that these dreams were more vivid than later visions, but I have suggested that they were more readily assimilated and recalled owing to the longer time one remained awake in bed and, equally important, due to the relative absence of noise, light, and other distractions during the dead of night. ‘Then hath thy soule the least incumbrance’, wrote the English moralist Francis Quarles of the interval following ‘the end of thy first sleep’.^44^ The fact is that today we quickly lose, i.e. fail to recall, the vast number of nocturnal visions upon awakening in the morning and rising from bed in a well-lit room to prepare for the day’s activities. ‘Like a morning dream’, wrote John Dryden in 1679, ‘vanish’d in the business of the day’.^45^ Although a modern estimate posits that ten per cent of our lives are devoted to dreaming, with the average person experiencing between one and two hundred thousand dreams in a lifetime, the overwhelming majority of these are forever lost.^46^ The author is correct that common wisdom attached greater ‘truth’ to later dreams^47^ but that does not appear to have affected the impact of nocturnal visions following an individual’s first sleep, as any number of examples attest. The Lancashire doctor Richard Kay wrote, for instance, in 1737, ‘I have dreamed dreams that when I have awoke out of them they have, even in the dark and silent night, brought me upon my knees and deeply humbled me’.^48^ Yet more revealing is the complaint voiced by the anonymous author of *The Art of Thriving: or, the Way to Get and Keep Money* (London, 1674): ‘What a ‘shame it is to spend half one’s lifetime in dreams and sleeps; leave your bed thereof when first sleep hath left you, lest custom render your body sluggish, or (which is worse) your mind a cage of unclean thoughts’.^49^ This quotation alone should be sufficiently dispositive to allay Boyce’s scepticism regarding segmented sleep in general and the impact of dreams in particular.

Apart from suggesting the possibility of ‘other models of early modern sleep’, Boyce concludes, ‘Constructing models of early modern night [sleep*?*] is by nature a tentative, limited and ambiguous process’.^50^ Indeed, he employs the word ‘might’ on upwards of forty occasions, in addition to relying upon a variety of other equivocal terms, such as ‘apparently’, ‘unclear’, and ‘perhaps’. He frequently minces words, including in instances that he himself cites, when evidence points to the prevalence of biphasic sleep. ‘Depositions and diaries’, he writes at one point, ‘support the existence of nocturnal conversations: however they confirm that people did indeed wake up in the night, but not necessarily that this was part of a common pattern of “first’” and “second sleep”’.^51^ His analysis in this instance and others calls to mind what the historian David Hackett Fischer has labelled the fallacy of the *double-reversing generalisation*: ‘It is a species of interpretative bet-hedging, which in an extreme form becomes no interpretation at all but a maze of mutual qualifications’.^52^

In the absence of advancing a conclusion upon a systematic review of hundreds of textual references to segmented sleep that are easily accessible, Boyce seeks, however subtly at times, to instil doubt and uncertainty based on parsing a tiny fraction of potential examples, excerpted chiefly from ‘Sleep We Have Lost’. One of which, a quotation from John Locke, due to its ambiguity, I stopped citing several years ago.^53^

Otherwise, I am puzzled by the author’s scepticism regarding other examples that he has selected. There is insufficient space to respond to all these instances, but it is impossible to ignore several of the most egregious inaccuracies. The author contends, for instance, that in my 2001 article I used several sources ‘that describe distinctly unusual circumstances’. He then proceeds to dissect at length the relevance of quotations from the authors William Davenant, William Baldwin, George Fidge, and Ogier de Gombauld.^54^ Each of these examples, however ‘unusual some’ of the circumstances in Boyce’s view (the sources after all are works of literature designed to appeal to contemporaries), clearly attest to the common experience of segmented sleep. Likewise, it is hard to imagine how the meaning of a quotation from George Wither – ‘I say at mid-night when thous wak’st from sleeepe’ – could be any clearer.^55^

Boyce, at one point, questions the meaning of Noel Taillepied’s reference to ‘about midnight when a man wakes from his first sleep’.^56^ We are informed by Boyce that the sleeper could have been awakened by the appearance of a ghost rather than seeing a ghost *only after* the sleeper has awakened naturally, which was Taillepied’s obvious meaning. Boyce sows further confusion in his analysis of a quotation from Andrew Boorde urging people when they awaken from their ‘first sleep’ to take the opportunity to urinate if they feel the need*:* ‘And whan you do wake of your fyrste slepe make water yf you fele your bladder charged’.^57^ Boyce misinterprets Boorde’s advice by writing ‘that people in the early modern period might have woken periodically by the need to urinate’. No doubt some occasionally did, but that is not the meaning of the quotation, which takes for granted that people naturally awakened in the middle of the night.

Boyce again confuses cause and effect by asserting that ‘the legal depositions that Ekirch cites refer to individuals rising from bed for a specific purpose’.^58^ Although many individuals, after awakening from their ‘first sleep’, expected to perform chores before taking their ‘second sleep’, people did not deliberately awaken for the specific purpose of performing tasks. Their deeds were designed to put to use their normal period of wakefulness. For women, these occasions typically included unskilled chores that required minimal light. Mary Collier’s poem, for instance, alludes to tasks performed after awakening at night (‘Often at midnight, from our bed we rise.’).^59^ Comparable is *Piers Plowman’s* description of female peasants: ‘They themselves also suffer much hunger, / and woe in wintertime, and waking up nights / To rise on the bedside, to rock the cradle, / Also to card and comb wool, to patch and to wash, / To rub flax and reel yarn and peel rushes’.^60^

In another instance, the author invokes the work of Handley,^61^ who graciously wrote me several years ago that her book, *Sleep in Early Modern England*, ‘makes plentiful reference to segmented sleep as a dominant pattern, though one (as you rightly note) that is not uniform’.^62^ As for the book written by Gabriele Klug (now Schichta), which Boyce cites, I could find nothing in the book to support any degree of ambiguity in the ‘linguistic meanings’ of ‘first’ and ‘second sleep’ – or anything else relevant to my research. Boyce quotes a personal message from Klug, dated 23 November 2020, in which she states that she found ‘some matching references’ to a “first sleep’ but none to a ‘second sleep’ or an intervening period of wakefulness in German texts written between 1150 and 1350. Whether Klug in her research was actively searching for references to segmented sleep is unclear, as is the likelihood that she knew of this pattern of slumber at the time. Instead, not unlike one of Boyce’s own conjectures, she assumed that nocturnal sleep in medieval Germany was continuous and that references to ‘first sleep’ merely reflected the ‘common knowledge’ that ‘sleep before midnight is deeper and “better”’ than sleep after midnight, which is to say not segmented into two intervals separated by a period of wakefulness.^63^

Other German texts that do refer to segmented sleep are listed on my website, including Grimmelshausen’s *Simplicius Simplicissimus* (1668), which refers directly to the protagonist’s ‘first sleep’.^64^ Martin Luther, to take another example, noted in a letter, ‘Almost every night when I wake up, the devil is there and wants to dispute with me. I have come to this conclusion. When the argument that the Christian is without the law and above the law doesn’t help, I instantly chase him away with a fart’. Are we to conclude that Luther was not referring to the interval of wakefulness between his ‘first’ and ‘second’ sleep?^65^

On page 99 of his article, Boyce writes, ‘Ekirch’s collection of segmented sleep references online includes an excerpt from a letter from Erasmus to Johann Choler, dated August 1535: "I accomplished this work, without being able to give it all the care it should have taken, but [during] hours of the afternoon, walking, while my families ate, sometimes in bed, while waiting for the second sleep”’. ‘Ekirch does not, however, note that at this point in Erasmus’ life, he was experiencing health problems, notably chronic pain caused by gout, which interfered with his sleep’. I was not previously aware that Erasmus suffered from occurrences of gout during much of his later life. Whether or not it was chronic is unclear. Likely on the August night in 1635, his gout, a painful inflammation that most often occurs in the big toe, was in remission inasmuch as he wrote in the same letter of ‘taking a walk, generally in the afternoon’.^66^

The relevance of a quotation from Sir James Chrichton-Browne from 1891 in which he recommends a ‘first sleep’ of eight hours is unclear, apart from the fact that it refers to a continuous period of sleep toward the end of the nineteenth century.^67^ Boyce writes, ‘First sleep is being used here to describe a first phase of sleep of an hour or two before midnight that is thought to be particularly beneficial, with no evidence that this is necessarily followed by a period of wakefulness’. In fact, Chrichton-Browne’s analysis is closely in accordance with my argument pertaining to the transformation of segmented sleep toward the late 1800s, which by then had become virtually continuous for most persons, as I discuss at length in ‘The Modernization of Western Sleep’.^68^

There is no contradiction, alleged by Boyce, in writing that ‘families rose from their beds’ during the hiatus of wakefulness to perform diverse activities, whereas other persons, I have pointed out, elected on some nights to remain in bed to pray, meditate, reflect upon dreams, engage in sex, or converse with a bedmate if one happened to be awake.^69^ As for the Saenredram print, I used it in two of my publications to suggest activity at night.^70^ I have never cited it as proof of segmented sleep, and I am surprised that Boyce has chosen to analyse it at such length.

Two final observations. It is not in the least remarkable, as I have recognised from the very beginning of my research, that the expression ‘first sleep’ could have alternate meanings: for example, ‘the first sleep of Adam’; ‘it was his first sleep in two days’; and ‘first, sleep’. Identifying such instances is not a matter of painstaking deconstruction as other historians would doubtless acknowledge. Why then does Boyce dilate upon this issue at such length? If it is to suggest that I have not been cognizant of similar references with different contexts and meanings – none of which have I ever quoted or cited other than those relating directly to segmented sleep^71^ – perhaps he could point to any errors in this regard. The expression ‘second sleep’, suffice to say, was also liable to alternate meanings, which naturally depended on the context of such instances.

A corollary is Boyce’s suggestion that ‘reading early modern texts with the assumption that segmented sleep was a routine and widespread practice might lead to misinterpretation, or rather, restricted interpretation of the evidence’. To be sure, he hastens to note, ‘This is not to argue that Ekirch arrived at his views on segmented sleep before his analysis of the copious evidence he provides. But it is to say that carefully re-examining the texts Ekirch presents might reveal new interpretations that do not rely on the segmented sleep model’.^72^ To be clear as to the correct chain of causation, only after a period in which I came across a growing number of references in primary sources to biphasic sleep did I gradually become convinced, to my great surprise, of their meaning and prevalence. I have ‘carefully’ conducted this historical research off and on for nearly forty years, yet Boyce nonetheless implies, however delicately, that I ‘might’ not have been sufficiently sensitive to the documentary evidence.

Boyce’s concern is ironic. No doubt the principal reason that it has taken so many years for this earlier pattern of human sleep to be recognised is that historians, myself included, have instinctively assumed nocturnal slumber to have been monophasic throughout human history. But to read that assumption back into the past is anachronistic. It conflates the present and the past by concluding distant patterns of human behaviour on the basis of modern populations. Further, I invite Boyce to provide a sufficiently persuasive corpus of references, drawn from primary sources, to early modern individuals who either slept through the night or made statements indicating that seamless sleep – or some unknown variant – was the predominant pattern of pre-industrial slumber in the Western world.

## Footnotes

1

Niall Boyce , ’Have we lost sleep? A Reconsideration of segmented sleep in early modern England’, Medical History, 67, 2 (2023), 91–108.37525459 10.1017/mdh.2023.14PMC10404514

2
*’Sleep We Have Lost: Pre-industrial Slumber in the British Isles’, The American Historical Review, CV, no.2 (April 2001), 343–387; At Day’s Close: Night in Times Past* (New York: W.W. Norton & Company, 2005), 261–323.

3

A. Roger Ekirch , ‘The Modernization of Western Sleep: Or, Does Insomnia Have a History?’ Past & Present, 226 (2015), 154–5; https://sites.google.com/vt.edu/roger-ekirch/sleep-research/segmented-sleep

4 ‘Modernization of Western Sleep’, *op. cit.* (note 3), 154.

5
*Ibid.*, 149–92.

6 ’Have we lost sleep?’, *op. cit*. (note 1), 93; Janine Rivière , Dreams in Early Modern England (New York: Taylor and Francis, 2017), 146 .

7 Aubrey, *Miscellanies on Various Subjects…* (London: A. Betteswoth, 1721); *At Day’s Close*, *op. cit.* (note 2), 300–1; https://sites.google.com/vt.edu/roger-ekirch/sleep-research/segmented-sleep. For early modern medicines and recipe books, see Sasha Handley , Sleep in Early Modern England (New Haven, CT: Yale University Press, 2016), 60–7.

8 ‘Have we lost sleep?’, *op. cit*. (note 1), 100, 104, 107.

9
*At Day’s Close*, *op. cit.* (note 2), *passim*, esp. 300-392; https://sites.google.com/vt.edu/roger-ekirch/sleep-research/segmented-sleep.

10

Bacon, Francis (1561–1626), The Works of Francis Bacon, Baron of Verulam, Viscount St. Alban, And Lord High Chancellor of England (London: A. Millar, 1765) I, 428.

11

Patrick J. Gallacher , ‘The Summoner’s Tale and Medieval Attitudes Towards Sickness’, Chaucer Review, xxi (1986), 211 n.6.

12

Lauren Joubert , Popular Errors, trans. Gregory David de Rocher (Tuscaloosa, ALA: University of Alabama Press, 1999), 112–3; https://sites.google.com/vt.edu/roger-ekirch/sleep-research/segmented-sleep. See also, for example, *At Day’s Close*, *op. cit.* (note 2), 308, 310.

13
https://sites.google.com/vt.edu/roger-ekirch/sleep-research/segmented-sleep.

14
https://sites.google.com/vt.edu/roger-ekirch/sleep-research/segmented-sleep. See also *At Day’s Close*, *op. cit.* (note 2), 271–2, 302.

15 Handley, *Sleep in Early Modern England*, *op. cit.* (note 7), 60–7; *At Day’s Close*, *op. cit.* (note 2), 271.

16 ‘Modernization of Western Sleep’, *op. cit.* (note 4), 156–7, 190–2.

17
https://vtx.vt.edu/articles/2022/01/clahs-roger-ekirch-outstanding-faculty

18 ‘Have we lost sleep?’, *op. cit*. (note 1), 97–98.

19

Paul Bohannan , ‘Concepts of Time among the Tiv of Nigeria’, Southwestern Journal of Anthropology, 9, 3 (1953), 253 ; Paul and Laura Bohannan , Three Source Notebooks in Tiv Ethnography (New Haven, CT: Human Relations Area File, 1958), 357 .

20

A.R. Ekirch , ‘Segmented Sleep in Preindustrial Societies’, Sleep, 36, 3 (2016), 715–610.5665/sleep.5558PMC476336526888454.

21

David R. Samson et al., ‘Segmented Sleep in a Nonelectric, Small-scale Agricultural Society in Madagascar’, American Journal of Human Biology, 29, 4 (2017), 29 10.1002/ajhb.2297928181718.

22

A. Thevet , The Peculiarities of French Antarctica, Otherwise Called (French) America, the Islands Discovered in our Times (Paris: Maisonneuve et cie, 1878), 147–9.

23

H. Wickham ,‘Notes on the Soumoo or Woolwa Indians, of Blewfields River, MosquitoTerritory’, Journal of the Anthropological Institute of Great Britain and Ireland, 24 (1895), 201.

24 Hattie Garlick, ‘The truth about ‘split sleeping’, *The Telegraph*, 9 January 2023. See also Foster’s earlier comment in Stephanie Hegarty, ‘The Myth of the Eight-Hour Sleep’, *BBC News Magazine*, 22 February 2012, https://www.bbc.com/news/magazine-16964783.

25 ‘Have we lost sleep?’, *op. cit*. (note 1), 96–97.

26 Thomas A. Wehr et al., ‘Conservation of Photoperiod-responsive Mechanisms Humans’, *American Journal of Physiology* (1993), 265; Thomas A. Wehr , ‘A ‘clock for all seasons’ in the human brain’, in R. M. Buijs et al. (eds), Hypothalamic Integration of Circadian Rhythms (Amsterdam: Elsevier, 1996), 319–40; Wehr, ‘The impact of changes in nightlength (scotoperiod) on human sleep’, in F. W. Turek and P. C. Zee (eds), *Regulation of Sleep and* Circadian Rhythms (New York: CRC Press, 1999), 263–85; Natalie Angier, ‘Modern life suppresses ancient body rhythm’, *New York Times*, 14 March 1995; personal communications with Thomas Wehr, 23, 31 December 1996.

27
*At Day’s Close*, *op. cit.* (note 2), 100–111.

28 ‘Have we lost sleep?’, *op. cit*. (note 1), 97.

29
*At Day’s Close*, *op. cit.* (note 2), 303.

30 ‘Have we lost sleep?’, *op. cit*. (note 1), 98.

31
*At Day’s Close*, *op. cit.* (note 2), 263–299.

32 Robert Sanderson Diary, St. John’s College, Cambridge, 12 November 1729, 30 November 1726, 4 January 1728.

33 ’Have we lost sleep?’, *op. cit*. (note 1), 99.

34
*Ibid*.

35
*Ibid.*, 94.

36
*Ibid*., 91.

37

M. Louis , Lettres sur la Certitude des Signes de la Mort (Paris: M. Lambert, 1752).

38

Eugène Sue , ‘The Wandering Jew’, The New World, 9, 30 (1844), 680.

39
https://sites.google.com/vt.edu/roger-ekirch/sleep-research/segmented-sleep.

40
*Ibid.*

41
*Ibid.*; *At Day’s Close*, *op. cit.* (note 2), 306.

42 ‘Modernization of Western Sleep’, *op. cit.* (note 4), 169.

43 ‘Have we lost sleep?’, *op. cit*. (note 1), 94-96.

44

Francis Quarles , Enchiridion: Containing Institutions, Divine Contemplative. Practicall. Moral Ethicall. Oeconomicall. Politicall (London: R.F. 1644), ch. 54.

45

John Dryden and Nathaniel Lee , Oedipus (London: R. Bentley and Magnes, 1679), 14 . See also John Whaley , ‘To Miss A.W. a Very Young Lady’, in A Collection of Original Poems and Translations (London: John Whaley, 1745), 257 ; Alexander B. Grosart , *The Complete Works in Verse and Prose of Samuel Daniel*, ed., 5 vols. (London: n.p.,1885–96), 2, 45 ; Nahum Tate , Brutus of Alba: or, The Enchanted Lovers (London: E.F., 1678), 6; *At Day’s Close, op. cit.* (note 2), 310–23.

46 ‘Sleep We Have Lost’, *op. cit.* (note 2), 385.

47 ‘Have we lost sleep?’, *op. cit*. (note 1), 94-95.

48 ‘Sleep We Have Lost’, *op. cit.* (note 2), 375.

49
*The Art of Thriving: or, the Way to Get and Keep Money …* (London: n.p. 1674).

50 ’Have we lost sleep?’, *op. cit*. (note 1), 108.

51
*Ibid*., 107.

52

David Hackett Fischer , Historians’ Fallacies: Toward a Logic of Historical Thought (New York: Oxford University Press, 1970), 125.

53 ‘Have we lost sleep?’, *op. cit*. (note 1), 105.

54
*Ibid*., 101–102.

55
*Ibid*, 105.

56
*Ibid*., 98–99.

57
*Ibid*., 106.

58
*Ibid*., 305–306.

59
*At Day’s Close*, *op. cit.* (note 2), 305–306.

60
*Ibid*.

61 ‘Have we lost sleep?’, *op. cit*. (note 1), 93–94.

62 Personal communication from Sasha Handley, 29 June 2020.

63 ‘Have we lost sleep?’, *op. cit*. (note 1), 94.

64
https://sites.google.com/vt.edu/roger-ekirch/sleep-research/segmented-sleep.

65 ‘Modernization of Western Sleep’, *op. cit.* (note 4), 152–3.

66 ‘Have we lost sleep?’, *op. cit*. (note 1), 99.

67
*Ibid*., 104.

68 ‘Modernization of Western Sleep’, *op. cit.* (note 4), 161-2.

69 ‘Have we lost sleep?’, *op. cit*. (note 1), 106.

70
*Ibid*., 107.

71
*Ibid.*, 102.

72
*Ibid.*, 94.

